# Expression Analysis of the Prolific Candidate Genes, *BMPR1B*, *BMP15,* and *GDF9* in Small Tail Han Ewes with Three Fecundity (*FecB* Gene) Genotypes

**DOI:** 10.3390/ani8100166

**Published:** 2018-09-28

**Authors:** Jishun Tang, Wenping Hu, Ran Di, Qiuyue Liu, Xiangyu Wang, Xiaosheng Zhang, Jinlong Zhang, Mingxing Chu

**Affiliations:** 1 Key Laboratory of Animal Genetics and Breeding and Reproduction of Ministry of Agriculture, Institute of Animal Science, Chinese Academy of Agricultural Sciences, Beijing 100193, China; tjs157@163.com (J.T.); pinkyhoho@163.com (W.H.); dirangirl@163.com (R.D.); liuqiuyue@caas.cn (Q.L.); xiangyu_wiggle@163.com (X.W.); 2 Institute of Animal Husbandry and Veterinary Medicine, Anhui Academy of Agricultural Sciences, Hefei 230031, China; 3 Tianjin Institute of Animal Sciences, Tianjin 300381, China; zhangxs0221@126.com (X.Z.); jlzhang1010@163.com (J.Z.)

**Keywords:** candidate gene, *FecB* genotype, tissue expression, sheep

## Abstract

**Simple Summary:**

As important prolific candidate genes, *BMPR1B, BMP15,* and *GDF9* may affect the lambing performance of sheep. Therefore, regarding the three *FecB* genotypes of Small Tail Han (STH) sheep (FecB BB, FecB B+, and FecB ++), this study explored the gene expression characteristics of different tissues using reverse transcription PCR (RT-PCR) and real-time quantitative PCR (qPCR). The results showed that *BMPR1B*, *BMP15*, and *GDF9* expression differed between the selected tissues, with all being highly expressed in the ovaries. Further analysis indicated that there was no significant difference in *BMPR1B* expression among the three *FecB* genotypes, but both *GDF9* and *BMP15* had the highest expression in FecB B+. As for other non-ovarian tissues, expression also varied. This study is relevant to understanding the high prolificacy of the STH breed.

**Abstract:**

The expression characteristics of the prolific candidate genes, *BMPR1B*, *BMP15*, and *GDF9*, in the major visceral organs and hypothalamic–pituitary–gonadal (HPG) axis tissues of three *FecB* genotypes (FecB BB, FecB B+, and FecB ++) were explored in STH ewes using RT-PCR and qPCR. The results were as follows, *BMPR1B* was expressed in all FecB BB genotype (Han BB) tissues, and *GDF9* was expressed in all selected tissues, but *BMP15* was specifically expressed in the ovaries. Further study of ovarian expression indicated that there was no difference in *BMPR1B* expression between genotypes, but the FecB B+ genotype (Han B+) had greater expression of *GDF9* and *BMP15* than Han BB and FecB ++ genotype (Han ++) (*p* < 0.05, *p* < 0.01). *BMP15* expression was lower in the ovaries of Han BB than in Han ++ sheep, but the reverse was shown for *GDF9*. The gene expression in non-ovarian tissues was also different between genotypes. Therefore, we consider that the three genes have an important function in ovine follicular development and maturation. This is the first systematic analysis of the tissue expression pattern of *BMPR1B*, *BMP15,* and *GDF9* genes in STH sheep of the three *FecB* genotypes. These results contribute to the understanding of the molecular regulatory mechanism for ovine reproduction.

## 1. Introduction

Understanding of the genetics of female fertility is of particular importance for economic production of meat sheep. Earlier work [1] found that between 74% and 96% of the economic value of genetic progress under selection was attributable to increases in litter size, which is ultimately influenced by ovulation rate. However, the vast majority of sheep breeds around the world are non-prolific breeds. Effectively increasing the litter size of sheep has been an urgent scientific challenge for a long time. Since the beginning of the 1980s, researchers have searched for the major genes related to the prolificacy of sheep [2]. Until now, three kinds of major candidate genes have been studied, including those with known mutations, those with known genetic modes, as well as those that need to be further validated [3]. Among them, the bone morphogenetic protein receptor 1B (*BMPR1B*), bone morphogenetic protein 15 (*BMP15*), and growth differentiation factor 9 (*GDF9*) are genes with known target mutations that have been investigated in relation to litter size [4,5,6,7,8,9]. BMPR1B, also known as activin receptor-like kinase 6 (ALK6), is an important transmembrane receptor protein that is involved in the signal transduction of bone morphogenetic protein 2 (BMP2), bone morphogenetic protein 4 (BMP4), bone morphogenetic protein 6 (BMP6)/Vg-related protein 1 (Vgr1), bone morphogenetic protein 7 (BMP7)/osteogenic protein 1 (OP1), growth differentiation factor 5 (GDF5), and bone morphogenetic protein 15 (BMP15) [10]. BMPR1B has a great influence on cumulus cell expansion, ovulation cycle, and skeletal system development. Early studies found that there is a mutation locus (A746G), named *FecB* by the International Committee on Sheep and Goat Genetics, in the coding region of the *BMPR1B*, which results in the amino acid change from glutamine (Glu) to arginine (Arg) at position 249 (Q249R) [11,12]. According to previously published literature, the FecB mutation has an additive effect on enhancement of the ovine ovulation number and litter size, such that one copy of the FecB mutation may increase the ovulation number by 1.5 and the litter size by 1, and two copies by 3 and 1.5, respectively [11,13].

BMP15 and GDF9, which both belong to the transforming growth factor β (TGFβ) superfamily, are oocyte-derived growth factors and can affect the ovulation rate of sheep in a dose-sensitive manner [14,15]. Recently, in animal models with autosomal mutations and knockout genes, *BMP15* has been reported as a critical gene that has great influence on the ovulation rate and litter size of mammals [14,16]. Many studies have revealed that *BMP15* and *GDF9* are mainly expressed in oocytes. BMP15 binds to specific receptors of the granulosa/sheath cell membrane surrounding oocytes [10,17], while GDF9 regulates follicular growth and differentiation, facilitating granulosa cell (GC) proliferation and maintaining the stability of the follicular microenvironment [18]. Recently, researchers found that the type I receptors located on granulosa cells (GC), such as BMPR1B, interact with BMP15 and GDF9, which results in the phosphorylation of downstream Smad signaling molecules, which, in turn, activates Smad-dependent and -independent signaling pathways, and regulates the transcription of downstream target genes. 

Research has also found that the mutated *BMPR1B* (*FecB*) gene is widely distributed in Asian sheep breeds, including Booroola Merino sheep [11], Indian Garole sheep [19], Indonesian Javanese Thin Tail sheep [19], Kendrapada sheep [20], Chinese Hu sheep [21], and STH sheep [22]. The STH sheep, an important source of both fibre and meat in China, is an endemic polytocous breed with year-round estrus and precocious puberty. In addition, the STH sheep also has comparatively excellent characters, such as crude feed tolerance, rapid growth, and good meat quality, etc. Therefore, STH sheep provide an ideal model breed to explore the molecular genetic mechanisms related to hyperprolificacy in certain breeds [23]. Recently published research showed that the STH sheep population can be divided into three *FecB* genotypes, by applying the TaqMan probe method. Frequencies of genotype BB, genotype B+, and genotype ++ were identified as 0.154–0.667, 0.273–0.692, and 0–0.333, respectively [24]. To date, many studies on the expression of these three genes in the tissues of different animals have been reported [16,24,25,26,27]. However, there have been few studies on the tissue expression profiles or quantitative analyses in FecB carrier and noncarrier STH sheep. In particular, expression during the follicular phase is of interest. Herein, we report the *BMPR1B*, *BMP15*, and *GDF9* expression levels of the three genotypes based on FecB in the major visceral organs, the HPG axis, and other tissues, including the oviduct and uterus in STH ewes, thus elucidating the genetic mechanism controlling high fecundity in certain breeds of sheep.

## 2. Material and Methods

### 2.1. Selection and Treatment of Experimental Sheep

Multiparous healthy ewes (approximately 2.5 years old and average weight of 60 kg) were selected from the nucleus herd of STH sheep in Shandong, China and fostered at the Sheep & Goat Breeding Farm of Tianjin Institute of Animal Sciences (Tianjin, China). All ewes were provided with high quality hay and clean water available *ad libitum.* The experimental ewes were divided into the three *FecB* genotypes using the TaqMan probe technique based on the FecB mutation including a homozygous mutant (FecB BB genotype, named Han BB), heterozygote mutant (FecB B+ genotype, named Han B+), and wild-type (FecB ++ genotype, named Han ++). All ewes were subjected to estrus synchronization administration of progesterone (CIDR device, InterAg Co., Ltd., Hamilton, New Zealand) for 12 days. Then, three healthy STH sheep from each of the three *FecB* genotypes were chosen for further analyses.

All experimental procedures mentioned in the present study were approved by the Science Research Department (in charge of animal welfare issue) of the Institute of Animal Sciences, Chinese Academy of Agricultural Sciences (IAS-CAAS) (Beijing, China). Ethical approval was given by the animal ethics committee of IAS-CAAS (No. IASCAAS-AE-03, 12 December 2016).

### 2.2. Sample Collection

The tissue samples were collected 45–48 h after CIDR removal, based on preliminary test observations. During this period, the developing follicles were at their largest, but have not yet ovulated. Fourteen tissue samples from the heart, liver, spleen, lung, kidney, brain, cerebellum, hypothalamus, pituitary, ovary, oviduct, uterus, adrenal gland, and duodenum were obtained from sheep of the three *FecB* genotypes. The tissue samples were collected within 30 min of euthanasia. Fresh samples were frozen in liquid nitrogen in 2 mL RNase-Free tubes (Thermo Fisher Scientific, Waltham, MA, USA) immediately and stored at −80 °C in the laboratory.

### 2.3. Total RNA Extraction and cDNA Synthesis

Tissue RNA was extracted from the 14 tissues using a total RNA extraction kit for animal tissue (Tiangen, Beijing, China) and Trizol (Invitrogen Inc., Carlsbad, CA, USA) was used to dissolve the tissues. The quantity and quality of total RNA were monitored by 1.5% agarose gel electrophoresis and ultraviolet spectrophotometry (UV-1201, Shimadzu, Kyoto, Japan), respectively. Then, the RNAs were stored at −80 °C until use.

The first strand of cDNA was prepared following the instructions of the PrimeScript^TM^ RT Reagent Kit (TaKaRa Bio Inc., Dalian, China). The reaction program was as follows. 37 °C for 15 min, followed by 85 °C for 5 s, with a total volume of 20 μL which contained 1 μg of total RNA, 1 μL of PrimeScript RT Enzyme Mix I, 1 μL of Oligo dT Primer, 1 μL of random 6-mers, 4 μL of 5× PrimeScript Buffer (for Real Time), and 12 μL of RNase-free water. Prior to storage at −80 °C, the cDNA quality was evaluated by housekeeping gene (*β-actin*) amplification, and then the reverse products were stored at −20 °C until use.

### 2.4. Primer Design

Primers were designed with Premier 3.0 (version 4.1.0) online software (http://primer3.ut.ee/). Primer sequences of *BMPR1B*, *BMP15*, *GDF9*, and *β-actin* were selected from GenBank (http://www.ncbi.nlm.nih.gov/) for RT-PCR and qPCR analyses. Previously published literature also provided a reference for the primer sequence of *BMP15* for PCR [28]. All primers were synthesized by the Beijing Tianyi Biotechnology Co., Ltd. (Beijing, China). The housekeeping gene (*β-actin*) was used as an internal control to normalize the threshold cycle (Ct) values. Primers are detailed in Table 1.

### 2.5. Exploration of Gene Amplification Parameters

To explore the optimal amplification cycles for *BMPR1B*, *BMP15*, and *GDF9* in RT-PCR, seven cycle parameters, 26, 28, 30, 32, 34, 36, and 38, were set to amplify the ovarian tissue cDNA by PCR.

### 2.6. RT-PCR Program and Amplification System

**Figure 1:**
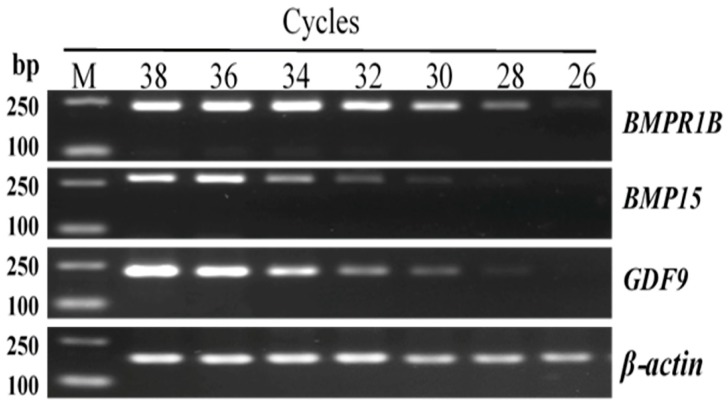
Amplification results of *BMPR1B, BMP15*, and *GDF9* in different reaction cycles. M: DL2000 DNA marker.

The reverse transcribed cDNA was used for the RT-PCR analysis. The volume of the amplification system of RT-PCR was 20 μL, including 10 μL of 2 × PCR Master Mix (Biomed, Beijing, China), 0.5 μL of 10 mmol/L primer (forward and reverse), 1.0 μL of cDNA, and 8 μL of ddH_2_O. The amplification program was as follows: initial denaturation at 95 °C for 5 min; followed by the optimal cycles (34, 34, and 36 cycles each for *BMPR1B*, *BMP15*, and *GDF9*) of denaturation at 95 °C for 30 s (see Figure 1); annealing for 30 s; and extension at 72 °C for 60 s, with a final extension at 72 °C for 5 min, and then the PCR product was stored at 4 °C.

### 2.7. qPCR

#### 2.7.1. System and Program for Real-Time qPCR Analyses

Tissue qPCR was performed in triplicate along with the negative controls (H_2_O used as template) using the Roche Light Cycler^®^480 II. The qPCR amplification mixture was 20 μL, containing 10 μL of SYBR^®^ Premix ExTaq II (TaKaRa Bio Inc., Dalian, China), 0.8 μL of primers (forward and reverse), 2 μL of cDNA, and the rest being ddH_2_O. The qPCR program is as follows, with the initial denaturation at 95 °C for 5 min, followed by 40 cycles of denaturation at 95 °C for 5 s and 60 °C for 30 s. The gene expression data were normalized to the *β-actin* gene. After amplification, the melting curve was analyzed.

#### 2.7.2. Establishment of the Standard Curve

One microliter of cDNA was pipetted from each sample. Then, eight gradient concentrations of cDNA were mixed and diluted: 1-, 2-, 4-, 8-, 16-, 32-, 64-, and 128-fold. *BMPR1B*, *BMP15*, *GDF9*, and *β-actin* were quantified by qPCR using this diluted cDNA as templates. The standard curves (Supplementary File 1) of the target and housekeeping genes were drawn as the C_t_ value (0–40) against the cDNA concentration (natural logarithm).

### 2.8. Data Statistics

The relative gene expression levels were calculated by the 2^−ΔΔCt^ method [29,30]. Statistical analyses were carried out using SPSS 22.0 software (IBM Armonk, NY, USA). The levels of gene expression were analyzed for significant differences with one-way analysis of variance (ANOVA), followed by the Fisher’s least significant difference (LSD) test as a multiple comparison test [31]. All experimental data are shown as mean ± SEM. Statistical significance was taken as *p* < 0.05. 

## 3. Results

### 3.1. RNA Extraction and cDNA Synthesis

Total RNA samples were analyzed using 1.5% agarose gel electrophoresis (U = 150 V; I = 240 mA). Three bands (representing 28S, 18S, and 5S) were detected—the 28S band was brighter than the 18S band, and the 5S band was unclear. The OD260 nm/OD280 nm ratios (1.8–2.0) of the samples RNA were all 1.9 to 2.0, which showed that the extracted total RNA was of sufficient purity with no contamination or degradation. Therefore, these tissue RNAs could be used in the follow-up experiment.

### 3.2. Exploration of the Amplification Parameters

In principle, to explore the optimal amplification parameters, it is important that the luminance of the gene amplification band remains unchanged with the increase in reaction cycles. As seen in Figure 1, the selective cycles of *BMPR1B, BMP15*, and *GDF9* were 34, 36, and 36, respectively.

### 3.3. Tissue-Specific Expression Analysis of BMPR1B, BMP15, and GDF9 

**Figure 2:**
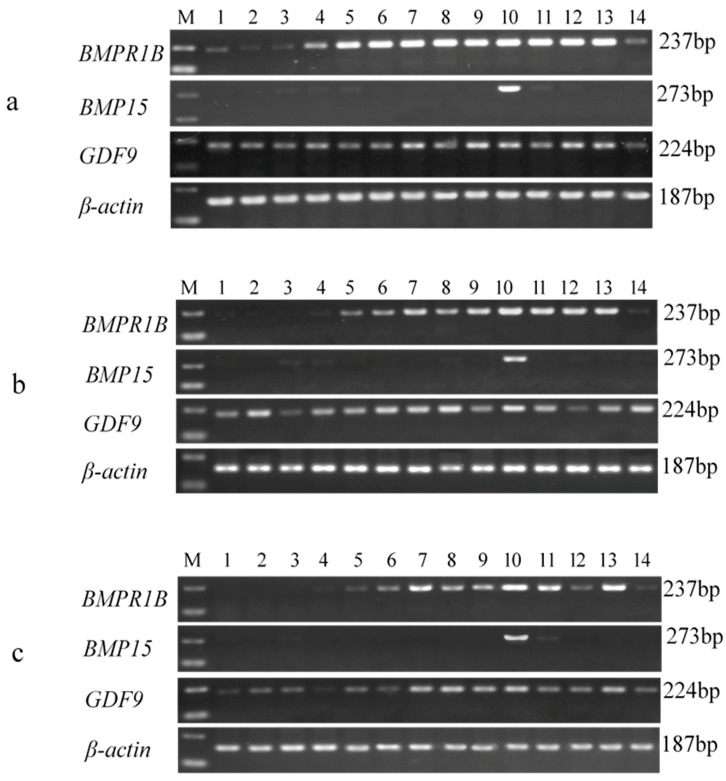
Tissue-specific expression analysis of *BMPR1B*, *BMP15*, *GDF9,* and *β-actin* genes in three genotypes of STH sheep. Han BB (**a**), Han B+ (**b**), and Han ++(**c**). 1–14: heart, liver, spleen, lung, kidney, brain, cerebellum, hypothalamus, pituitary, ovary, uterus, oviduct, adrenal gland, and duodenum, respectively. M: DL2000 DNA marker.

The results of the RT-PCR amplification showed that the fragment lengths of the amplification products of *BMPR1B*, *BMP15*, *GDF9*, and *β-actin* were consistent with their theoretical lengths, identified by agarose gel electrophoresis (1.5%). As seen in Figure 2, the β-actin gene, as the reference gene, was successfully expressed in all 14 tissues of STH sheep of the three *FecB* genotypes. *BMPR1B* was expressed in all selected tissues of the Han BB sheep, and was highly expressed in seven tissues (brain, cerebellum, hypothalamus, pituitary, ovary, uterus, and adrenal gland) of the Han B+ and Han ++ sheep. *BMP15* was highly expressed in the ovaries of sheep of all three *FecB* genotypes. Additionally, *GDF9* was expressed in all 14 tissues of all sheep.

### 3.4. Expression Levels of BMPR1B, BMP15, and GDF9 in the HPG Axis 

It is very difficult to quantitatively analyze gene expression using RT-PCR. Therefore, the expression levels of *BMPR1B*, *BMP15*, and *GDF9* in the HPG axis (hypothalamus, pituitary, and ovary), uterus, and oviduct tissues of the STH sheep were measured by qPCR.

**Figure 3:**
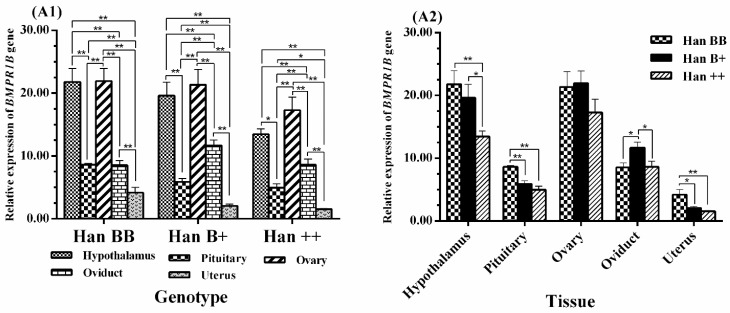
Comparison of expression of *BMPR1B* among three *FecB* genotypes (**A1**) and among tissues (**A2**). Means with different superscripts are significantly different. The significant results with a *p*-value lower than 0.01, 0.05 are giving two asterisks (**) and one asterisk (*), respectively. Nonsignificant results are not giving any asterisks.

As shown in Figure 3, *BMPR1B* was expressed in five tissues with the three *FecB* genotypes, with the highest level being in the ovaries (*p* < 0.01), followed by the hypothalamus, with no significant difference between the two. However, the expression level of *BMPR1B* in the hypothalamus was significantly higher than those of the pituitary, oviduct, and uterus (*p* < 0.01), and expression levels in the pituitary and oviduct were significantly higher than those of the uterus (*p* < 0.01, *p* < 0.05). In Han B+ and Han ++ sheep, the expression level of *BMPR1B* was significantly higher in the oviduct than in the pituitary (*p* < 0.01). There was no significant difference in the *BMPR1B* ovarian expression levels between genotypes, but its expression in the hypothalamus of Han BB and Han B+ was higher than that in Han ++ sheep (*p* < 0.01, *p* < 0.05). *BMPR1B* expression levels in the pituitary and uterus were significantly higher in Han BB than in Han B+ and Han ++ sheep (*p* < 0.01, *p* < 0.05). However, *BMPR1B* expression in the oviduct was significantly higher in Han B+ than in Han BB and Han ++ sheep (*p* < 0.05).

**Figure 4:**
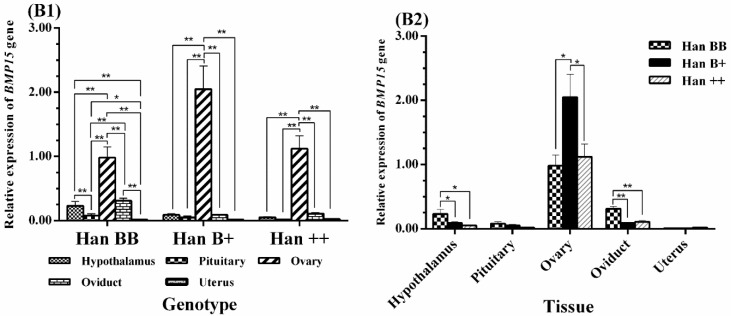
Comparison of expression of *BMP15* among three *FecB* genotypes (**B1**) and among tissues (**B2**). Means with different superscripts are significantly different. The significant results with a *p*-value lower than 0.01, 0.05 are giving two asterisks (**) and one asterisk (*), respectively. Nonsignificant results are not giving any asterisks.

The results of the analysis are shown in Figure 4, where among the five tissues examined, *BMP15* was highly significantly expressed in the ovaries (*p* < 0.01) and presented in trace levels in non-ovarian tissues. Additionally, the expression levels of *BMP15* in the hypothalamus and oviduct of Han BB sheep were all much higher than those of the pituitary and uterus (*p* < 0.01), and expression in the pituitary was higher than in the uterus (*p* < 0.05). The tissue expression profiles of the three genotypes of sheep were analyzed, and the expression level of *BMP15* in the ovaries of Han B+ sheep was significantly higher than in Han BB and Han ++ sheep (*p* < 0.05), and its expression in the ovaries of Han BB was lower than in Han ++ sheep; however, there was no significant difference between the two. Furthermore, the expression levels of *BMP15* in the hypothalamus and oviduct tissues were much higher in Han BB than in Han B+ and Han ++ sheep (*p* < 0.05, *p* < 0.01).

**Figure 5:**
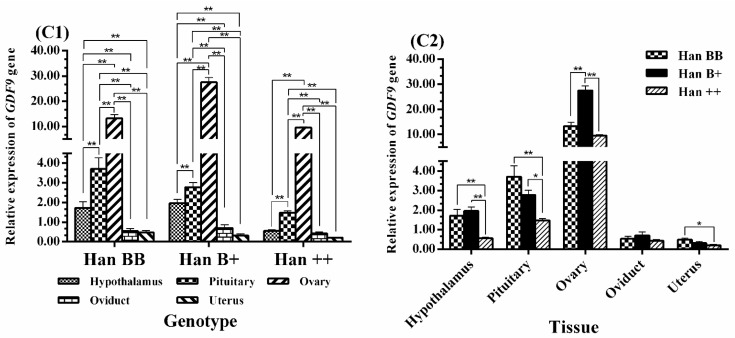
Comparison of expression of *GDF9* among three *FecB* genotypes (**C1**) and among tissues (**C2**). Means with different superscripts are significantly different. The significant results with a *p*-value lower than 0.01, 0.05 are giving two asterisks (**) and one asterisk (*), respectively. Nonsignificant results are not giving any asterisks.

Figure 5 clearly shows that the expression levels of *GDF9* in the ovaries of sheep of the three *FecB* genotypes showed a similar pattern to *BMP15*, with its expression level being significantly higher in the pituitary than in the hypothalamus, oviduct, and uterus (*p* < 0.01). In addition, the expression level of *GDF9* was significantly higher in the hypothalamus of Han BB and Han B+ sheep than in other tissues including the oviduct and uterus (*p* < 0.01). In the comparison between genotypes, the expression level of *GDF9* in the ovaries was much higher in Han B+ than in Han BB and Han ++ sheep (*p* < 0.01 or *p* < 0.05), and its expression in the ovaries was higher in Han BB than in Han ++ shepp, but there was no significant difference between the two (*p* >0.05). In addition, *GDF9* expression in the hypothalamus and pituitary was significantly higher in Han BB and Han B+ than in Han ++ sheep (*p* < 0.01, *p*< 0.05), and its expression in the uterus was much higher in Han BB than in Han ++ sheep (*p* < 0.05).

## 4. Discussion

### 4.1. BMPR1B Expression

Previous reports have found that *BMPR1B* plays the same role as the protein kinase activity of serine or threonine, which has an important influence on ovarian biological function [32]. *BMPR1B* expression has previously been detected in the reproductive tissues, brain, skeletal muscle, and kidney tissues of sheep [11]. In this study, *BMPR1B* was shown to be highly and widely expressed in tissues, including the ovary and hypothalamus, in the three *FecB* genotypes of STH sheep. In Chinese Merino sheep, during the estrus period *BMPR1B* was shown to be expressed in the ovary, ear, spinal cord, pituitary, bone, uterus, hypothalamus, kidney, skeletal muscle, and oviduct tissues, which indicates that its function is diverse and that it plays a critical role in the ovaries in addition to bone formation [33]. However, the expression levels of *BMPR1B* in the ovarian tissues of the three *FecB* genotypes in Chinese Merino sheep ewes showed no significant difference [33]. Subsequently, *BMPR1B* expression in the follicles of multiparous Hu sheep was found to be significantly higher than that of uniparous Hu sheep, which indicated that it may influence the ovulation rate by regulating the BMP/Smad signal pathway and some related cytokines [34]. In addition, some researchers have found that *BMPR1B* has no tissue-specific expression in whole tissues, and it was also shown to have a high expression level in the ovaries of the Jining Grey goat and Liaoning Cashmere goat, implying that it not only exerts a great influence on the process of bone formation, but also has a potential role in ovarian development and even in the process of reproduction. However, no significant difference in expression was shown between these two goat breeds, which suggested that the *BMPR1B* may not contribute to the high fecundity of goats [35]. Similarly, it has also been speculated that differences in reproductive performance may be ascribed to the structural differences in *BMPR1B* between Lezhi black and Tibetan goats [36].

On the basis of the similarity of expression levels of ovarian *BMPR1B* in the three FecB genotypes, we propose that *BMPR1B* plays an important biological role in the lambing performance of STH sheep through mutation rather than expression level. Early studies found that granulosa cells (GC) may be inhibited by *BMPR1B*, but the inhibition effect of the mutated *BMPR1B* was weaker for GC. Sheep carrying the *FecB* mutation exhibit higher ovulation numbers [11], which may be the cause of the different litter sizes of sheep in the three *FecB* genotypes. Moreover, the expression differences in *BMPR1B* in non-ovarian tissues of the HPG axis of sheep of the three *FecB* genotypes were not consistent with the results from goat breeds [35]. We infer that the expression profile described above may have a species discrepancy or the different timings of the sampling may have influenced the results. Regarding *BMPR1B* expression in non-ovarian tissues, especially in the oviduct, previous research has suggested that its expression in the ampullary region of the bovine oviduct is significantly higher than in the isthmus area and may affect the normal physiological function of oviduct epithelial cells [37]. Therefore, we needed to explore whether the difference in expression of *BMPR1B* in non-ovarian tissues influences the ovulation rate and litter sizes of STH sheep of the three *FecB* genotypes. According to the analysis of function annotation and tissue-specific expression, especially regarding the high level of expression in the ovaries, the biological function of *BMPR1B* appears to be diverse. It can be speculated that *BMPR1B* exerts a pivotal influence on ovarian function, follicular development, and maturity.

### 4.2. BMP15 Expression

BMP15, which is secreted by oocytes, is an indispensable signaling molecule for normal follicular development [38] and can play a critical regulatory role in the growth and differentiation of early oocytes [39,40]. Studies have found that it can activate the mRNA transcription of PFKP (phosphofructokinase, platelet type) and lactate dehydrogenase A (LDHA), which are required for granulosa cell glycolysis [41]. Likewise, it can promote the expression of kit ligand mRNA in granulosa cells [42] and antagonize the apoptosis of cumulus cells [43]. Expression of *BMP15* was first detected in mouse oocytes [44]. Subsequently, other researchers have found it to have high specific expression in human oocytes [17], as well as in rats [14], pigs [25], and sheep [45]. In this study, *BMP15* was shown to be highly expressed in the ovaries, with trace expression in five other tissues (spleen, lung, kidney, hypothalamus, and oviduct), which is consistent with the results in Hu sheep [46]. Previous research indicated that BMP15 can downregulate the follicle stimulating hormone (FSH) effect, promote cell proliferation, and ensure the growth and maturity of oocytes by inhibiting the expression of the FSH receptor [35]. Therefore, *BMP15*, based on its high expression in the ovary, may be an important factor in maintaining numerous follicular developments.

In this study, *BMP15* expression was lower in the ovaries of Han BB sheep than in Han B+ and Han ++ sheep. From previous studies, it is well-known that the expression level of *BMP15* in the ovaries of *FecB* mutant homozygous (FecB BB genotype) Booroola Romney sheep is significantly lower than that in the wide-type (FecB ++ genotype), which suggests that the higher ovulation rate of BB genotype sheep is due to a decrease in the *BMP15* mRNA level and an earlier onset of luteinizing hormone (LH)-responsiveness in granulosa cells (GC) [45]. The receptor concentration of synthetic estrogen, inhibin, and follicle stimulating hormone (FSH) in follicular granulosa cells decreases due to whole or partial deletion of *BMP15* function, which further leads to lower levels of FSH that maintain the follicular growth. Therefore, it can be inferred that ewes with the FecB mutant have a high ovulation rate, which may be responsible for the low expression of *BMP15* in the ovaries of Han BB sheep. However, some studies have also concluded that *BMP15* is a key gene in the high fecundity of goats based on results where *BMP15* expression in the hircine ovary was shown to be significantly higher in a polytocous breed than in a monotocous breed [35,47]. These studies suggest that *BMP15* has a substantial regulatory effect on the ovulation rates of sheep and goat [48,49]. The high level expression of *BMP15* in the ovaries of Han B+ sheep, implies that *BMP15* may in addition to its demonstrated function in the ovary of *FecB* B+ genotype STH sheep, have other influences on reproduction. For *BMP15*, in regard to the different expression levels in non-ovarian tissues, we hypothesize that unclear effects related to production or interrelationships with other genes could lead to this expression profile. Generally speaking, the high expression of *BMP15* in the ovaries indicates that it plays a key role in the growth and maturation of oocytes. However, its expression level may be negatively correlated with the litter sizes of STH sheep.

### 4.3. GDF9 Expression 

GDF9 is secreted by oocytes and performs pivotal regulation in follicular growth and differentiation by means of the paracrine pathway [50]. A previous study reported that GDF9 promotes the expression of hyaluronan synthase 2 and urokinase plasminogen activator (UPA) in the cumulus oocyte complex (COC) and activates the cumulus expansion [51]. *GDF9* and its protein have been expressed in many mammal oocytes via in situ hybridization and immunohistochemical technologies [52,53,54]. *GDF9* was also detected in GC [18] and non-ovarian tissues, including the hypothalamus pituitary and testis [26]. Particularly in Hu sheep, it is expressed in 10 tissues (hypothalamic, pituitary, ovary, oviduct, uterus, heart, liver, spleen, lung, and kidney tissues) [55], which implies that *GDF9* has a wide range of biological effects. In our study, *GDF9* was expressed in all tissues, of which the ovaries showed the highest expression, which indicates that it plays an important part not only in reproductive organs, but also in other organs and tissues.

Additionally, previous studies revealed that *GDF9* expression in the ovaries is significantly higher in prolific Hu sheep than in non-prolific Hu sheep, indicating that it is a differentially expressed gene and could also affect the BMP/Smad metabolic pathway to regulate the litter sizes of sheep [34]. Previous research has revealed that *GDF9* can exert a synergistic effect with FSH, BMP15, and other hormones or growth factors during follicular development [18]. In this study, the *GDF9* expression levels in the ovaries were higher in STH sheep with Han BB and Han B+ genotypes than in those with Han ++ genotype, which indicates that *GDF9* expression may affect the lambing performance of different *FecB* genotypes of STH sheep. However, this is an inconsistent conclusion as the *GDF9* expression level in the ovaries was not significantly different between FecB carrier and noncarrier Indian sheep [56]. It was also found that the expression levels of *GDF9* in the HPG axis tissues of the Jining Grey goat (multiparous breed) and Liaoning Cashmere goat (uniparious breed) are not significantly different [35]. These different findings may be the result of differences between species or breeds and the timeliness of sampling. As a consequence, *GDF9* may implement a similar function in these tissues to promote oocyte maturity and further affect the ovulation rate in STH sheep. Therefore, we concluded that *GDF9* function is complex and plays a positive regulatory role in the differentiation and development of antral follicles, eventually possibly increasing the ovulation rate and litter size of STH sheep with the *FecB* mutation.

## 5. Conclusions

This study found that *BMPR1B*, *BMP15*, and *GDF9* are highly expressed in the ovaries of three *FecB* genotypes of STH sheep, which indicates that they may play pivotal functions in the ovine ovaries and promote follicular growth and maturation. The *BMPR1B* expression level may not differ significantly in the ovaries of STH ewes of the three genotypes, while *BMP15* and *GDF9* genes have an important biological function in the ovary and influence the reproduction performance of *FecB* B+ genotype STH sheep. This is the first systematic analysis of tissue expression patterns of the three genes tissue expression pattern in *FecB* genotypes of STH sheep.

## Figures and Tables

**Table 1:** Primers of studied genes.

Method	Gene Name	Primer Sequence (5′→3′)	Length (bp)	Tm (°C)	Accession No.
qPCR primer	*BMPR1B*	F: 5′-TGACGGACCTATACACCACA-3′ R: 5′-GTACCGAGGTCTGGCTTCTT-3′	121	60	NM_001142888.2
	*BMP15*	F: 5′-TGTTGGGCAAAAGCTCTGGA-3′ R: 5′-GCCATGCCACCAGAACTCAA-3′	106	60	NM_001114767.1
	*GDF9*	F: 5′-AACAGACGCCACCTCTACAA-3′ R: 5′-CACGATCCAGGTTAAACAGCA-3′	124	60	NM_001009431.1
	*β-actin*	F: 5′-ACCCAGCACGATGAAGATCA-3′ R: 5′-GTAACGCAGCTAACAGTCCG-3	97	60	NM_001009784.1
RT-PCR primer	*BMPR1B*	F: 5′-GGGTTCTACGACTCCGCTTC-3′ R: 5′-GGTTACTTTCAGGCCCATCAT-3	237	60	NM_001142888.2
	*BMP15*	F: 5′-GGGTTCTACGACTCCGCTTC-3′ R: 5′-GGTTACTTTCAGGCCCATCAT-3′	273	62	NM_001114767.1
	*GDF9*	F: 5′-TAGTCAGCTGAAGTGGGACA-3′ R: 5′-AGCCATCAGGCTCGATGGCC-3′	224	61	NM_001009431.1
	*β-actin*	F: 5′-ACCCAGCACGATGAAGATCA-3′ R: 5′-GTAACGCAGCTAACAGTCCG-3′	187	61	NM_001009784.1

## Supplementary Materials

The following are available online at http://www.mdpi.com/2076-2615/8/10/166/s1. Supplementary File 1: Standard Curve of target gene.
