# Supplementary material for: Expression Analysis of the Prolific Candidate Genes, BMPR1B, BMP15, and GDF9 in Small Tail Han Ewes with Three Fecundity (FecB Gene) Genotypes
Source: Animals (Basel). 2018 Sep 28;8(10):166. doi: 10.3390/ani8100166 (PMC6210785; doi:10.3390/ani8100166)

Supplement Fig. S1-S4:   Standard Curve of target gene

Fig.s1**:** Standard Curve of *β-actin* gene

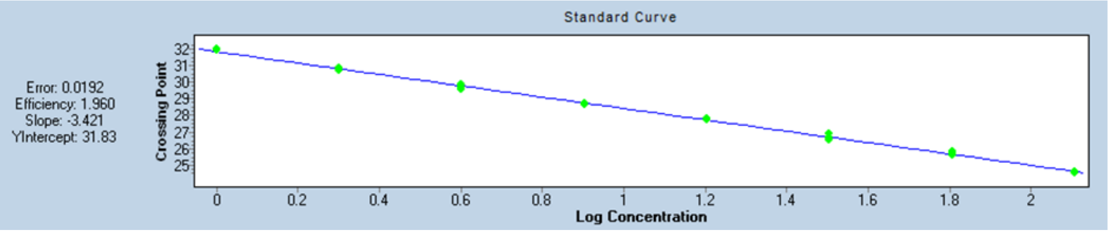

Fig.s2**:** Standard Curve of *BMPR1B* gene

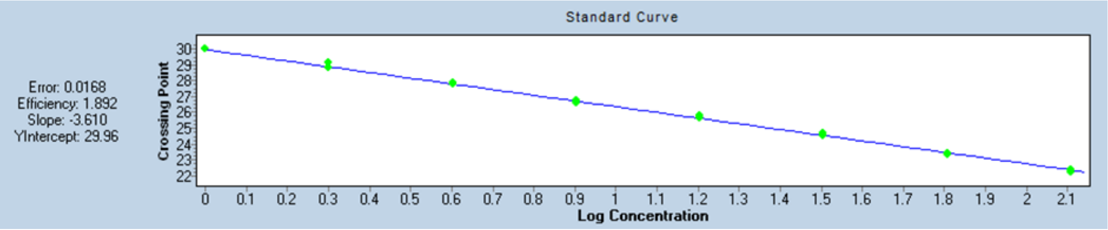

Fig.s3： Standard Curve of *BMP15* gene

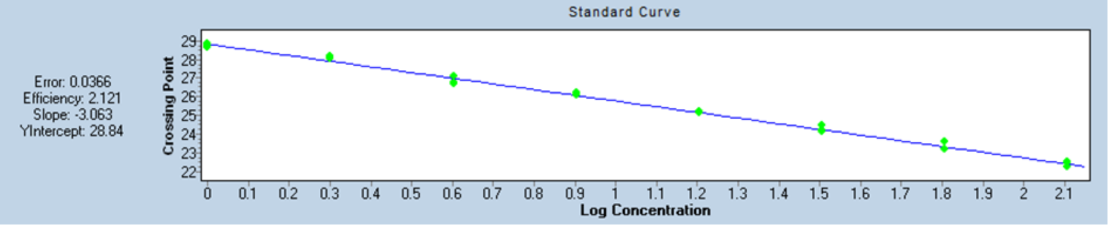

Fig. s4： Standard Curve of *GDF9* gene

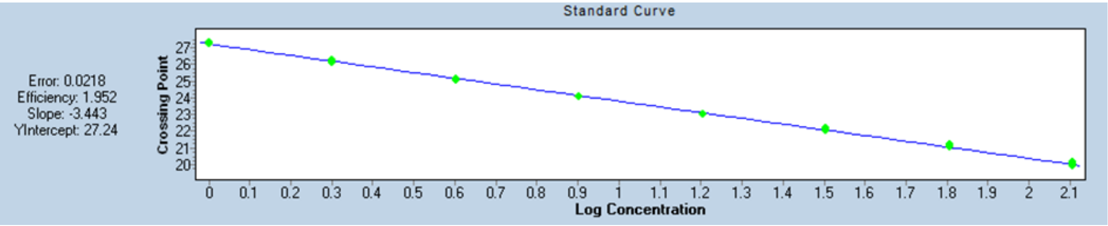

Supplement: Supplementary file 1 [file animals-08-00166-s001.pdf]
